# Rehabilitation Therapists Have Better Job Satisfaction Than Nurses in a Tertiary Rehabilitation Healthcare Setting: A Cross-Sectional Study

**DOI:** 10.7759/cureus.73018

**Published:** 2024-11-04

**Authors:** Abdullah H Bin Zarah, Majed Al Mohareb

**Affiliations:** 1 Management/Administration Department, Sultan Bin Abdulaziz Humanitarian City, Riyadh, SAU; 2 Human Capital Department, Sultan Bin Abdulaziz Humanitarian City, Riyadh, SAU

**Keywords:** healthcare, job satisfaction, nurses, occupation, rehabilitation, therapists

## Abstract

Introduction: It is an established fact that job satisfaction of medical staff is a crucial factor that affects healthcare quality and plays a major role in the retention of qualified staff.

Aim: This study aims to assess the occupational satisfaction of nurses and physiotherapists in a tertiary rehabilitation institution, holding a bed capacity of 511 beds, by utilizing the Job Satisfaction Survey (JSS) questionnaire.

Methods: This cross-sectional survey was conducted at a tertiary rehabilitation hospital between August and November 2022. The study targeted full-time nurses and rehabilitation therapists working in the institution using an English questionnaire focusing on the Job Satisfaction Survey (JSS) that was sent electronically to all eligible staff. The JSS questionnaire assessed job satisfaction and consisted of 36 items in nine domains of job satisfaction, which included pay, promotion, supervision, benefits, rewards, operational procedures, coworkers, work itself, and communications.

Findings: The response rate was 64.43% among nurses and 64.93% among therapists. The satisfaction rate was higher among therapists (34.41%) compared to nurses (26.25%). The overall job satisfaction mean score was significantly higher among therapists compared to nurses at 149.28±27.12 compared to 143.14±31.67, respectively. This was also observed in the pay domain (13.06±4.06 versus 12.36±4.29), fringe benefits domain (12.77±4.89 versus 11.90±4.82), and coworkers' domain (15.55±3.67 versus 14.71±4.17), while it was significantly higher among nurses compared to therapists when looking at the promotion domain at 14.27±3.52 versus 13.75±3.16, respectively. When doing regression analysis, the odds ratio (OR) was the highest among bachelor nurses and those with more than 40 years of age for therapists.

Conclusion: Rehabilitation therapists are more satisfied than nurses with the main satisfaction domains being financial benefits in the form of pay, fringe benefits, and contingency rewards. Age, gender, and work duration did not show any significant value in this study, and overall, the dissatisfied group for both rehabilitation therapists and nurses did not manifest any difference in most of the job satisfaction factors.

## Introduction

Job gratification and career contentment are key outcomes from a job that impact and influence the work ethic and service quality of all professionals. The healthcare sector is one of the most sensitive professional fields that involves dealing with lives, young and old alike, and hence, the quality of care delivered by professionals is of utmost importance for all, the employee and employer and the patient. It is an established fact that job satisfaction of medical staff is a crucial factor that impacts healthcare delivery and the performance of employees besides playing a major role in the retention of qualified staff [1]. Due to the sensitivity and nature of this field, this effect is seen to be even more pronounced among medical professionals who have direct and prolonged patient contact, e.g., nurses and rehabilitation therapists [2]. Besides it being a pleasant and positive emotional state that reflects a person's job performance and experience related to the work [3], job satisfaction is a key indicator that many health institutes are now striving to focus on. This is becoming a highlighting focus lately, in order to ensure high-quality healthcare delivery and the provision of the best environment, benefits, career initiatives, and objectives to incentivize employees to remain devoted assets to their workplace and organization [4,5].

Several studies have shown that medical staff's job satisfaction can be influenced by different factors, such as employees' age, income, shift work, years of experience, and training [4]. The medical field has a multidisciplinary healthcare force that may impact the health economy and patients' care [5]. However, due to its sensitive, energy- and time-consuming nature, practicing in a healthcare environment often generates stress and exhaustion, which can ultimately contribute to the dissatisfaction of the medical staff and the possibility of burnout [6].

Literature shows that medical staff job satisfaction is inversely related to the patients' direct contact [7]. This puts nurses and rehabilitation therapists under the real challenge of being the highest medical specialties with direct patient contact and raises the fundamental question about their job satisfaction. A local study has shown that physiotherapists from the governmental and private sectors are not fully satisfied with their jobs, which warrants more exploration to value the extent of this dissatisfaction [8]. Another study that was conducted among nurses in Saudi Arabia supports the same conclusion observed among physiotherapists [9].

The lack of data from specialized rehabilitation centers, where both disciplines are on the frontlines, warrants further exploring into the job satisfaction level of staff and the factors behind this dissatisfaction. Current literature lacks a focus on nurses and physiotherapists and is scarce, especially within the region.

Sultan Bin Abdulaziz Humanitarian City is a large rehabilitation hospital that has high occupancy and a fast turnover rate and manages patients with different disabilities and neuromuscular diseases covering both adult and pediatric patients. Nurses and therapists contribute to more than 45% of the total employees of this institution.

This study aims to assess the job satisfaction of nurses and physiotherapists in a tertiary rehabilitation institution, holding a bed capacity of 511 beds, by utilizing the Job Satisfaction Survey (JSS) questionnaire [10].

## Materials and methods

Study design and setting participants

This cross-sectional survey was conducted at Sultan Bin Abdulaziz Humanitarian City, Riyadh, Kingdom of Saudi Arabia (KSA), a tertiary rehabilitation hospital, between August and November 2022. The study targeted nurses and rehabilitation therapists working in the institution. The Institutional Review Board (IRB) of Sultan Bin Abdulaziz Humanitarian City issued approval 91-2023-IRB.

Inclusion and exclusion criteria

The study included all nurses and rehabilitation therapists working in the institution for more than six months and who were willing to complete the survey questionnaire. The survey excluded part-time employees or those recruited for less than six months.

Data collection

A questionnaire, in the English language, was designed with a cover letter, focusing on the Job Satisfaction Survey (JSS), and was sent electronically to all eligible nurses and rehabilitation therapists. Consent was taken from all participants who were made aware of their responses being recorded for study and research purposes. The participants were then encouraged to participate anonymously using a Microsoft Forms (Microsoft Corp., Redmond, WA) online survey creator, a part of Office 365.

Study tools and questionnaire

The questionnaire was designed with the first part being related to the sociodemographic characteristics of the participants, including age, nationality, gender, marital status, educational level, profession, years of experience, years of working in the hospital, and department (either nursing or rehabilitation department).

The second section assessed job satisfaction using the JSS questionnaire, which has 36 items that measure nine domains of job satisfaction, each assessed by four questions, which include pay, promotion, supervision, benefits, rewards, operational procedures, coworkers, work itself, and communications (Appendices).

The questionnaire has a 6-point Likert-type scale, with 6 indicating "very much agree" and 1 indicating "very much disagree" [10,11]. For the four-item subscales and the 36-item total score, satisfaction is represented by mean item responses (after reversing the negatively worded items) of 4 or higher. On the other hand, mean responses of 3 or lower indicate dissatisfaction, while mean scores between 3 and 4 reflect ambivalence. In terms of summed scores, the four-item subscales have a range of 4-24. Scores between 4 and 12 indicate dissatisfaction, scores between 16 and 24 indicate satisfaction, and scores between 12 and 16 represent ambivalence. For the 36-item total score, which can range from 36 to 216, the ranges are as follows: scores between 36 and 108 indicate dissatisfaction, scores between 144 and 216 indicate satisfaction, and scores between 108 and 144 indicate ambivalence.

All participants' completed forms were electronically archived for further assessment and analysis. Out of the total nursing staff of 402 nurses, 259 (64.43%) responded, and of a total of 381 rehabilitation therapists, 247 (64.83%) responded.

Statistical analysis

Data were analyzed using the Statistical Packages for the Social Sciences (SPSS) version 25 (IBM Corp., Armonk, NY). Descriptive analysis was done in the form of categorical data being presented as numbers and percentages and continuous variables as mean (standard deviation (SD)). Student's t-test was used to compare continuous variables, and the Chi-squared test was used to compare categorical variables. Odds ratios (ORs) (with a 95% confidence interval (CI)) were used for assessing variables that were considered to be associated with job satisfaction, and data was presented as a forest plot. P value less than or equal to 0.05 was considered to be statistically significant, and all interpretations were made accordingly.

## Results

Sociodemographic

A total of 506 staff participated in the survey. Out of them, 259 (51.19%) were nurses and 247 (48.81%) were rehabilitation therapists of different disciplines. The mean age of the nurses was significantly higher than the therapists, and the vast majority of the nurses (96.53%) were non-Saudis, while the majority of the therapists were Saudis (74.10%). Despite the fact of female gender predominance in both groups, the male gender was significantly higher among therapists compared to nurses at 36.84% and 24.71%, respectively. More therapists than nurses were seen in the unmarried category as single (55.47% versus 37.45%). The rate of participants with bachelor's degrees was significantly higher among nurses compared to therapists at 94.59% and 86.64%, respectively, while the rate of Master's degree holders was significantly higher among therapists (13.36%) compared to nurses (5.41%). More than 50% of the therapists had job experience of less than six years, compared to only 53.67% of nurses who had more than 10 years of job experience. On the other hand, the proportion of nurses who had more than 10 years of experience was significantly higher than that of therapists at 53.67% and 25.51%, respectively. When looking at employees' years working in the hospital, the proportion of nurses with more than 10 years of experience in the hospital was almost two times higher than therapists at 28.19% and 14.17%, respectively, as shown in Table 1.

**Table 1:** Baseline characteristics of nursing and rehabilitation staff who responded to the JSS JSS: Job Satisfaction Survey, SD: standard deviation

Variables	Categories	Total (N=506)	Nurses (n=259)	Therapist (n=247)	P value
Age (mean±SD)		34.60±7.38	37.41±7.27	31.66±6.29	<0.0001
Age (number (%))	21-30	200 (39.53)	43 (16.6)	157 (63.56)	<0.0001
	31-40	196 (38.74)	137 (52.9)	59 (23.89)	<0.0001
	Above 40	110 (21.74)	79 (30.5)	31 (12.55)	<0.0001
Nationality (number (%))	Saudi	194 (38.34)	9 (3.47)	185 (74.9)	<0.0001
	Non-Saudi	312 (61.66)	250 (96.53)	62 (25.1)	<0.0001
Gender (number (%))	Male	155 (30.63)	64 (24.71)	91 (36.84)	0.0031
	Female	351 (69.37)	195 (75.29)	156 (63.16)	0.0031
Marital status (number (%))	Single	234 (46.25)	97 (37.45)	137 (55.47)	<0.0001
	Married	261 (51.58)	155 (59.85)	106 (42.91)	0.0001
	Divorced/widowed	11 (2.17)	7 (2.70)	4 (1.62)	0.6583
Educational level (number (%))	Bachelor	459 (90.71)	245 (94.59)	214 (86.64)	0.0021
	Post-graduate	47 (9.29)	14 (5.41)	33 (13.36)	0.0032
Years of experience (number (%))	1-5 years	173 (34.19)	37 (14.29)	136 (55.06)	<0.0001
	6-10 years	131 (25.89)	83 (32.05)	48 (19.43)	0.0012
	Above 10 years	202 (39.92)	139 (53.67)	63 (25.51)	<0.0001
Years of working at the hospital (number (%))	1-5 years	272 (53.75)	114 (44.02)	158 (63.97)	<0.0001
	6-10 years	126 (24.9)	72 (27.8)	54 (21.86)	0.1228
	Above 10 years	108 (21.34)	73 (28.19)	35 (14.17)	0.0001
Departments (number (%))	Administration	26 (5.14)	17 (6.56)	9 (3.64)	0.1372
	Medical/paramedical	456 (90.12)	239 (92.28)	217 (87.85)	0.0954
	Other	24 (4.74)	3 (1.16)	21 (8.5)	0.0671

JSS

The overall job satisfaction mean score was significantly higher among therapists compared to nurses at 149.28±27.12 and 143.14±31.67, respectively. This was also observed in the pay domain (13.06±4.06 versus 12.36±4.29), fringe benefits domain (12.77±4.89 versus 11.90±4.82), and coworkers' domain (15.55±3.67 versus 14.71±4.17), while it was significantly higher in nurses compared to therapists when looking at the promotion domain at 14.27±3.52 and 13.75±3.16, respectively, as shown in Table 2.

**Table 2:** Total and per domain mean job satisfaction score among nursing and rehabilitation staff Overall satisfaction: satisfied: ≥144, ambivalent: 109-143, and dissatisfied: 36-108 Subdomain satisfaction: satisfied: ≥16, ambivalent: 13-15, and dissatisfied: 4-12 SD: standard deviation

Job satisfaction domains	Total	Nurses	Therapist	P value
	Score (mean (SD))	Score (mean (SD))	Score (mean (SD))	
Pay	13.01 (4.19)	12.63 (4.29)	13.06(4.06)	0.035
Promotion	14.06 (3.36)	14.27 (3.52)	13.75 (3.16)	0.031
Supervision	15.33 (3.42)	15.14 (3.56)	15.54 (3.27)	0.186
Fringe benefits	12.32 (4.87)	11.9 (4.82)	12.77 (4.89)	0.043
Contingency rewards	13.22 (5.57)	12.88 (5.47)	13.58 (5.66)	0.156
Operating conditions	13.99 (4.19)	13.74 (4.34)	14.25 (4.01)	0.171
Coworkers	15.12 (3.95)	14.71 (4.17)	15.55 (3.67)	0.016
Nature of work	18.14 (4.96)	17.89 (5.15)	18.4 (4.75)	0.245
Communication	15.56 (5.4)	15.39 (5.79)	15.72 (4.97)	0.492
Overall	131.42 (27.57)	143.14 (31.67)	149.28 (27.12)	0.020

Table 3 shows the rate of satisfaction among the two groups. The overall satisfaction rate was significantly higher among therapists (34.41%) compared to nurses (26.25%), with a P value of 0.046. However, the rates of employees with ambivalent feelings or dissatisfaction were comparable between the two groups with no significant difference. Concerning the job satisfaction domains, the rate of satisfaction per domain was in favor of the therapist for all the domains, with the difference in the rate being significant only in the fringe benefits at 32.39% and 24.32% for therapists and nurses, respectively. On the other hand, the rate of dissatisfaction per domain was higher among nurses compared to therapists, with the difference being significantly higher in the coworker's domain at 24.71% for nurses and 16.19% for therapists, with a P value of 0.017.

**Table 3:** Total and per domain job satisfaction rate among 259 nurses and 247 therapists

Job satisfaction domains	Nurses (satisfied) (number (%))	Therapist (satisfied) (number (%))	P value	Nurses (ambivalent) (number (%))	Therapist (ambivalent) (number (%))	P value	Nurses (dissatisfied) (number (%))	Therapist (dissatisfied) (number (%))	P value
Pay	70 (27.03)	72 (29.15)	0.97	70 (27.03)	81 (32.79)	0.15	119 (45.95)	94 (38.06)	0.072
Promotion	81 (31.27)	96 (38.87)	0.07	106 (40.93)	90 (36.44)	0.30	72 (27.8)	61 (24.7)	0.428
Supervision	124 (47.88)	134 (54.25)	0.15	101 (39.0)	83 (33.6)	0.20	34 (13.13)	30 (12.15)	0.740
Fringe benefits	63 (24.32)	80 (32.39)	0.044	63 (24.32)	53 (21.46)	0.44	133 (51.35)	114 (46.15)	0.242
Contingency rewards	82 (31.66)	97 (39.27)	0.073	47 (18.15)	45 (18.22)	0.98	130 (50.19)	105 (42.51)	0.083
Operating conditions	88 (33.98)	93 (37.65)	0.38	86 (33.2)	79 (31.98)	0.77	85 (32.82)	75 (30.36)	0.552
Coworkers	126 (48.65)	137 (55.47)	0.12	69 (26.64)	70 (28.34)	0.66	64 (24.71)	40 (16.19)	0.017
Nature of work	199 (76.83)	202 (81.78)	0.17	26 (10.04)	23 (9.31)	0.78	34 (13.13)	22 (8.91)	0.130
Communication	134 (51.74)	131 (53.04)	0.77	50 (19.31)	54 (21.86)	0.47	75 (28.96)	62 (25.1)	0.329
Overall	68 (26.25)	85 (34.41)	0.046	146 (56.37)	132 (53.44)	0.50	45 (17.37)	30 (12.15)	0.098

The correlation between satisfaction and dissatisfaction among nurses has shown a significant effect for the age groups tested, nationality, and gender, while years of working for nurses at the hospital were significantly in favor of <5 years of experience for the satisfied group only. This was also true for therapists for all different domains, as shown in Table 4.

**Table 4:** Overall satisfaction rate among nurses and therapists according to sociodemographic characteristics

Variables	Categories	Overall								
		Satisfaction		P value	Ambivalent		P value	Dissatisfied		P value
		Nurses (n=68) (number (%))	Therapist (n=85) (number (%))		Nurses (n=146) (number (%))	Therapist (n=132) (number (%))		Nurses (n=45) (number (%))	Therapist (n=30) (number (%))	
Age (number (%))	21-30	12 (17.65)	56 (65.88)	<0.001	19 (13.01)	84 (63.63)	<0.0001	12 (26.66)	17 (56.66)	0.0094
	31-40	37 (54.41)	22 (25.88)	<0.001	79 (54.10)	30 (22.72)	<0.0001	21 (46.66)	7 (23.33)	0.0421
	Above 40	19 (27.94)	7 (8.24)	<0.001	48 (32.87)	18 (13.63)	<0.0001	12 (26.66)	6 (20)	0.5110
	P value	<0.001	<0.001		<0.001	<0.001		0.028	0.028	
Nationality (number (%))	Saudi	1 (1.47)	63 (74.12)	<0.001	2 (1.36)	102 (77.27)	<0.0001	6 (13.33)	20 (66.67)	<0.0001
	Non-Saudi	67 (98.53)	22 (25.88)	<0.001	144 (98.63)	30 (22.73)	<0.0001	39 (86.67)	10 (33.33)	<0.0001
	P value	<0.001	<0.001		<0.001	<0.001		<0.001	<0.001	
Gender (number (%))	Male	12 (17.65)	34 (40)	<0.001	106 (72.6)	84 (63.64)	0.1094	33 (73.33)	21 (70)	0.5425
	Female	56 (82.35)	51 (60)	<0.001	40 (27.4)	48 (36.36)	0.1094	12 (26.67)	9 (30)	0.5313
	P value	0.001	0.009		<0.001	<0.001		<0.001	0.002	
Educational level (number (%))	Bachelor	65 (95.59)	72 (84.71)	0.029	139 (95.20)	117 (88.63)	0.0432	41 (91.11)	25 (83.33)	0.3130
	Post-graduate	3 (4.41)	13(15.29)	0.045	7 (4.80)	15 (11.37)	0.0431	4 (8.89)	5 (16.67)	0.3129
	P value	<0.001	<0.001		<0.001	<0.001		<0.001	<0.001	
Years of working at the hospital (number (%))	1-5 years	35 (51.47)	59 (69.41)	0.023	57 (39.04)	83 (62.87)	0.0001	22 (48.89)	16 (53.33)	0.708
	6-10 years	17 (25.01)	17 (20.01)	0.461	43 (29.45)	27 (20.45)	0.0848	12 (26.67)	10 (33.33)	0.537
	Above 10 years	16 (23.52)	9 (10.58)	0.032	46 (31.51)	22 (16.68)	0.0041	11 (24.44)	4 (13.34)	0.242
	P value	0.043	0.023		0.011	0.024		0.482	0.312	

When looking at the overall score for satisfied and dissatisfied nurses and therapists, therapists were significantly more satisfied, while nurses were more in the dissatisfied group when compared with therapists, although the difference was not statistically significant as shown in Table 3.

This study tested the most important factors that may affect satisfaction, namely, education, nationality, years of experience, gender, and age. When doing regression analysis, the odds ratio (OR) was the highest among nurses who had bachelor's degrees and therapists who were more than 40 years of age. However, none of the ORs had P values below 0.05, and thus, the results yielded were not significant. Therapists being Saudis and males had an odds ratio of more than 1, but again with no significant value, as shown in Table 5.

**Table 5:** Factors associated with job satisfaction among nurses and therapists OR: odds ratio, CI: confidence interval

Variables	Nurses				Therapists			
	Dissatisfied	Satisfied	OR (95% CI)	P value	Dissatisfied	Satisfied	OR (95% CI)	P value
Age < 40 years	30	47	1.11 (0.5-2.5)	0.79	24	76	2.11 (0.68-6.5)	0.21
Age ≥ 40 years	15	21			6	9		
Male	12	12	0.58 (0.23-1.46)	0.25	9	34	1.5 (0.63-3.8)	0.32
Female	33	56			21	51		
Experience ˂ 10 years	31	49	1.16 (0.5-2.6)	0.71	25	70	0.9 (0.3-2.8)	0.92
Experience ≥ 10 years	14	19			5	15		
Saudi	6	1	0.09 (0.01-0.83)	0.015	20	63	1.43 (0.58-3.5)	0.43
Non-Saudi	39	67			10	22		
Bachelor	41	65	2.11 (0.44-9.9)	0.43	25	72	1.10 (0.35-3.4)	0.53
Post-graduate	4	3			5	13		

## Discussion

Using JSS to assess nurses' and rehabilitation therapists' satisfaction rates, the study results have shown that the overall job satisfaction score among therapists compared to nurses was 149.28±27.12 and 143.14±31.67 respectively, indicating that rehabilitation therapists are more satisfied.

This was higher than what has been reported from similar institutions internationally from Greece and the USA, with the total mean score values at 128.3±20.5 and 133.1±27.9, respectively [12,13]. It was also much higher than a local study by Alkassabi et al. among physiotherapists who had shown a mean total score within the "ambivalent" category [8]. This high job satisfaction rate may reflect the good working environment at the hospital, especially when it was awarded as the best work environment in December 2014.

Both rehabilitation therapists and nurses had a high satisfaction rate, although it was higher among therapists, which is the same observation when looking at other international studies [14]. This could be related to the nature of work and no on-call duties being more in favor of rehabilitation therapists. Although nationality did not affect the satisfaction rate, therapists who had shown the highest satisfaction rate were predominantly Saudis. This could be explained by the cultural factor, where the physiotherapy profession is accepted culturally by Saudis more than the nursing profession.

Gender also did not show a significant difference in job satisfaction rate when regression analysis was down as shown in the forest plot, although males contribute to only one-quarter of nurses and one-third of rehabilitation therapists. This was similar to what has been found in Iran, where there was no significant difference between both genders in all job satisfaction dimensions [15], although a local study from Al-Madinah Al-Munawwarah that was conducted in a general hospital has shown that female nurses had higher satisfaction rates [16].

Job satisfaction did not correlate with years of experience, where half of the therapists who had a higher satisfaction rate had an experience of less than six years when compared with nurses for the same duration. There seems to be a negative correlation between job satisfaction for both disciplines with years of experience at the hospital. This is similar to other international studies, which could be explained by the fact that greater tenure can result in increased boredom and workload and, therefore, lower job satisfaction [17].

When comparing the satisfied employees with the dissatisfied ones for nurses and therapists, older age, Saudi nationality, and female gender were in favor of satisfaction, although the results for this were not significant. At the same time, years of working at the hospital had better satisfaction than dissatisfaction when working years at the hospital were less than or equal to five years [18].

Job satisfaction domains dissect the areas for employee satisfaction related to financial benefits, work environment, and supervision. Among the nine tested domains, there is a clear difference between the two disciplines where therapists are more satisfied with their pay, fringe benefits, and coworker domains. This reflects the city's human capital policy for payments and fringe benefits that are more in favor of therapists being less available and having more competition in the Saudi market [19].

The nurses in this study had a higher score in the promotion domain, which reflects the human capital policy adopted for nurses' promotion that provides a better chance for hard-working employees. In comparison, they had a lower score in the pay and fringe benefits domain as a result of more offers than demand and longer work experience in the hospital [20].

Regarding dissatisfaction, it was noticed to be greater in nurses than in therapists, being more obvious in the coworker domain, which possibly reflects the high working pressure and long working hours that may affect the relationship between nurses since it has been observed in this study that one-quarter of the studied nurses were not happy about their colleagues, in addition to the load of work and huge interaction between nurses when delivering patients care [21]. However, although only one-quarter of the nurses (26.25%) were satisfied, the study results showed statistics higher than what has been reported among nurses from India at 6.67% using the same survey questionnaire [22]. This could be explained by the fact that the nurse-to-patient ratio is better in this institution compared to the ratio in India [23].

The overall score for satisfied and dissatisfied nurses and therapists concludes that therapists are more significantly satisfied in most of the JSS domains. On the contrary, nurses are non-significantly dissatisfied with most of the JSS domains. This is reflected by the job nature of both disciplines since therapists had a clear and short duration patient commitment versus multiple and long job commitment by the nurses [24].

The value of each risk factor was measured through the strength of the association using the odds ratio, where it was found that education in the nurses and age of more than 40 years among therapists had the strongest association. However, it was not significant. On the other hand, Saudi nationality and male gender among therapists had the strongest association, but it did not have a significant odds ratio. Among nurses, experience of more than 10 years and age of more than 40 years did not have any impact on job satisfaction domains, while in therapists, a bachelor's degree and experience of more than 10 years did not reflect any role in job satisfaction.

This study has tested the most important factors that may affect job satisfaction, namely, educational level, nationality, years of experience, gender, and age, and has met its objectives of comparing results to preexisting literature within and beyond the region. The main satisfaction domains in this study are shown to be financial benefits in the form of pay, fringe benefits, and contingency rewards, a reflection of the human capital special payment ladder that is more in favor of this discipline in the Kingdom.

Age, gender, and work duration did not show any significant value in this study, which could be explained by the fact that the study cohort is heterogeneous in mean age, education, and work experience.

To justify the high job satisfaction rate observed in this study in comparison with general hospitals, nurses and rehabilitation therapists in rehabilitation institutes are not exposed to acute medical conditions and are more specialized and focused on chronic cases. In addition, they are not committed to frequent shifts and long working hours.

Limitations

Although this study covered almost 60% of those two disciplines in this institute, it cannot be ruled out that the remaining 40% may have negative opinions that may affect the overall satisfaction rate and bring factors related to dissatisfaction. The other limitation of this study was convincing employees to participate to reach a response rate of more than 64%.

Another limitation of the current study is the predominance of non-Saudis among nurses who participated in the study. Such findings might limit the generalizability of the results to Saudi nurses.

## Conclusions

This study concludes that rehabilitation therapists, in general, are more satisfied than nurses based on their nature of work since they are the main workforce in a rehabilitation institution with the main satisfaction domains being financial benefits in the form of pay, fringe benefits, and contingency rewards, which is a reflection of the human capital special payment ladder that is more in favor of this discipline in the Kingdom. Age, gender, and work duration did not show any significant value in this study, and overall, the dissatisfied group for both rehabilitation therapists and nurses did not manifest any difference in most of the job satisfaction factors. However, a good environment, supervision, and coworkers were seen to increase the overall satisfaction rate.

Further literature would help strengthen evidence specific to nurses and therapists and bridge the literature gap further. This study also highlights the need for health administrators and policymakers to ensure the installment and utilization of performance checks and employee satisfaction tools within all healthcare settings as key performance indicators (KPIs).

## Appendices

The Job Satisfaction Survey (JSS) questionnaire used in this study is shown in Table 6 (English) and Table 7 (Arabic).

**Table 6:** JSS English questionnaire JSS: Job Satisfaction Survey Job Satisfaction Survey (Paul E. Spector Department of Psychology University of South Florida, Copyright Paul E. Spector 1994, All rights reserved.)

Number	Job Satisfaction Survey						
	Please circle one number for each question that comes closest to reflecting your opinion about it.						
	Questions	Disagree very much	Disagree moderately	Disagree slightly	Agree slightly	Agree moderately	Agree very much
1	I feel I am being paid a fair amount for the work I do.	1	2	3	4	5	6
2	There is really too little chance for promotion at my job.	1	2	3	4	5	6
3	My supervisor is quite competent in doing his/her job.	1	2	3	4	5	6
4	I am not satisfied with the benefits I receive.	1	2	3	4	5	6
5	When I do a good job, I receive the recognition for it that I should receive.	1	2	3	4	5	6
6	Many of our rules and procedures make doing a good job difficult.	1	2	3	4	5	6
7	I like the people I work with.	1	2	3	4	5	6
8	I sometimes feel my job is meaningless.	1	2	3	4	5	6
9	Communications seem good within this organization.	1	2	3	4	5	6
10	Raises are too few and far between.	1	2	3	4	5	6
11	Those who do well on the job stand a fair chance of being promoted.	1	2	3	4	5	6
12	My supervisor is unfair to me.	1	2	3	4	5	6
13	The benefits we receive are as good as most other organizations offer.	1	2	3	4	5	6
14	I do not feel that the work I do is appreciated.	1	2	3	4	5	6
15	My efforts to do a good job are seldom blocked by red tape.	1	2	3	4	5	6
16	I find I have to work harder at my job because of the incompetence of the people I work with.	1	2	3	4	5	6
17	I like doing the things I do at work.	1	2	3	4	5	6
18	The goals of this organization are not clear to me.	1	2	3	4	5	6
19	I feel unappreciated by the organization when I think about what they pay me.	1	2	3	4	5	6
20	People get ahead as fast here as they do in other places.	1	2	3	4	5	6
21	My supervisor shows too little interest in the feelings of subordinates.	1	2	3	4	5	6
22	The benefit package we have is equitable.	1	2	3	4	5	6
23	There are few rewards for those who work here.	1	2	3	4	5	6
24	I have too much to do at work.	1	2	3	4	5	6
25	I enjoy my coworkers.	1	2	3	4	5	6
26	I often feel that I do not know what is going on with the organization.	1	2	3	4	5	6
27	I feel a sense of pride in doing my job.	1	2	3	4	5	6
28	I feel satisfied with my chances for salary increases.	1	2	3	4	5	6
29	There are benefits we do not have that we should have.	1	2	3	4	5	6
30	I like my supervisor.	1	2	3	4	5	6
31	I have too much paperwork.	1	2	3	4	5	6
32	I don't feel my efforts are rewarded the way they should be.	1	2	3	4	5	6
33	I am satisfied with my chances for promotion.	1	2	3	4	5	6
34	There is too much bickering and fighting at work.	1	2	3	4	5	6
35	My job is enjoyable.	1	2	3	4	5	6
36	Work assignments are not fully explained.	1	2	3	4	5	6

**Table 7:** Arabic JSS questionnaire JSS: Job Satisfaction Survey

	يرجى وضع دائرة حول رقم واحد من خيارات كل سؤال و الذي يكون الأقرب للتعبير عن رأيك حول هذا الموضوع.	لا أوافق بشده	لا أوافق بصوره متوسطه	لا أوافق قليلا	أوافق قليلا	أوافق بصوره متوسطه	أوافق بشده
1	أشعر أنني أتقاضى أجرا عادلا على العمل الذي أقوم به	1	2	3	4	5	6
2	حقيقةً هناك فرص قليله جدا للترقية في عملي .	1	2	3	4	5	6
3	إن مسؤولي المباشر كفؤ جدا في أدائه / أدائها للعمل	1	2	3	4	5	6
4	أنا غير راضٍ عن الامتيازات التي أحصل عليها .	1	2	3	4	5	6
5	عندما أقوم بعمل جيد أحصل على التقدير الذي يجب أن أحصل عليه عن ذلك العمل .	1	2	3	4	5	6
6	هناك عدة أنظمة وإجراءات تجعل القيام بالعمل الجيد أمراّ صعباّ	1	2	3	4	5	6
7	أنا (أرتاح) أحب الناس الذين أعمل معهم .	1	2	3	4	5	6
8	أشعر أحيانا أن عملي بلا معنى.	1	2	3	4	5	6
9	الاتصالات تبدو جيدة ضمن إطار هذه المؤسسة .	1	2	3	4	5	6
10	العلاوات قليلة جدا و متباعدة زمنيا .	1	2	3	4	5	6
11	الذين يؤدون عملهم بصورة جيدة يحظون بفرص جيدة للترقيه.	1	2	3	4	5	6
12	المسؤول المباشر عنى في العمل غير عادل معي.	1	2	3	4	5	6
13	الامتيازات التي نحصل عليها هي نفس الامتيازات في المؤسسات الأخرى.	1	2	3	4	5	6
14	لا اشعر بأن العمل الذي أقوم به يلقى التقدير.	1	2	3	4	5	6
15	جهودي المبذولة لكي أقوم بعمل جيد نادرا ما تكون دون جدوى أو (تذهب سدىً) .	1	2	3	4	5	6
16	وجدت بأنه علي إن اعمل بجد اكبر في عملي و ذلك لعدم كفاءة الأشخاص الذين اعمل معهم .	1	2	3	4	5	6
17	أحب الأعمال التي أقوم بها في عملي .	1	2	3	4	5	6
18	غايات هذه المؤسسة غير واضحة بالنسبة لي .	1	2	3	4	5	6
19	أشعر بعدم التقدير في المؤسسه عندما أفكر بالأجر الذي أتقاضاه منها.	1	2	3	4	5	6
20	يتقدم الناس وظيفيا في هذه المؤسسه بالسرعه التي يتقدمون بها في الأماكن أو المؤسسات الأخرى .	1	2	3	4	5	6
21	المشرف علي في العمل يعطي اهتماما قليلا لمشاعر العاملين المشرف عليهم .	1	2	3	4	5	6
22	أن حزمة الامتيازات التي نحصل عليها عادله.	1	2	3	4	5	6
23	المكافأات قليله للعاملين هنا .	1	2	3	4	5	6
24	هناك واجبات كثيرة في العمل .	1	2	3	4	5	6
25	أنا مستمتع بعملي مع الزملاء.	1	2	3	4	5	6
26	غالبا ما اشعر بأني لا اعرف ماذا يحدث في المؤسسة	1	2	3	4	5	6
27	أنا اشعر بالفخر عندما أؤدي عملي .	1	2	3	4	5	6
28	اشعر بالرضى عن الفرص المتاحة في زيادة الأجور .	1	2	3	4	5	6
29	هنالك فوائد من المفترض أن نحصل عليها و لكنها غير موجودة .	1	2	3	4	5	6
30	أنا أحب المشرف علي في العمل .	1	2	3	4	5	6
31	عندي الكثير من الأعمال الكتابية في العمل.	1	2	3	4	5	6
32	أنا أشعر بأن جهودي لا تكافئ بالطريقة الصحيحة التي يجب أن تكافئ بها.	1	2	3	4	5	6
33	أنا راض عن فرصي في الترقيه في العمل .	1	2	3	4	5	6
34	هناك الكثير من المشاحنات والشجار في العمل .	1	2	3	4	5	6
35	عملي ممتع .	1	2	3	4	5	6
36	الواجبات في العمل غير موضحة بصورة مفصله.	1	2	3	4	5	6

## References

[1] Khamlub S, Harun-Or-Rashid M, Sarker MA, Hirosawa T, Outavong P, Sakamoto J (2013). Job satisfaction of health-care workers at health centers in Vientiane Capital and Bolikhamsai Province, Lao PDR. Nagoya J Med Sci.

[2] Cohen B, Hyman S, Rosenberg L, Larson E (2012). Frequency of patient contact with health care personnel and visitors: implications for infection prevention. Jt Comm J Qual Patient Saf.

[3] Kalinowska P, Marcinowicz L (2020). Job satisfaction among family nurses in Poland: a questionnaire-based study. Nurs Open.

[4] Al-Dossary R, Vail J, Macfarlane F (2012). Job satisfaction of nurses in a Saudi Arabian university teaching hospital: a cross-sectional study. Int Nurs Rev.

[5] Fulmer IS, Ployhart RE (2013). "Our most important asset": a multidisciplinary/multilevel review of human capital valuation for research and practice. J Manag.

[6] De Hert S (2020). Burnout in healthcare workers: prevalence, impact and preventative strategies. Local Reg Anesth.

[7] El Mouaddib H, Sebbani M, Mansouri A, Adarmouch L, Amine M (2023). Job satisfaction of primary healthcare professionals (public sector): a cross-sectional study in Morocco. Heliyon.

[8] Alkassabi OY, Al-Sobayel H, Al-Eisa ES, Buragadda S, Alghadir AH, Iqbal A (2018). Job satisfaction among physiotherapists in Saudi Arabia: does the leadership style matter?. BMC Health Serv Res.

[9] Al Anazi M (2021). Work value and job satisfaction among Saudi nurses at major tertiary hospital. Saudi J Nurs Health Care.

[10] Spector PE (1985). Measurement of human service staff satisfaction: development of the job satisfaction survey. Am J Community Psychol.

[11] Spector PE (2022). Job satisfaction: from assessment to intervention.

[12] Tsounis A, Sarafis P (2018). Validity and reliability of the Greek translation of the Job Satisfaction Survey (JSS). BMC Psychol.

[13] Spector PE (1985). Measurement of human service staff satisfaction: development of the Job Satisfaction Survey. Am J Community Psychol.

[14] Fleury MJ, Grenier G, Bamvita JM (2017). A comparative study of job satisfaction among nurses, psychologists/psychotherapists and social workers working in Quebec mental health teams. BMC Nurs.

[15] Akbari M, Bagheri A, Fathollahi A, Darvish M (2020). Job satisfaction among nurses in Iran: does gender matter?. J Multidiscip Healthc.

[16] Al Juhani AM, Kishk NA (2006). Job satisfaction among primary health care physicians and nurses in Al-madinah Al-munawwara. J Egypt Public Health Assoc.

[17] Dobrow SR, Ganzach Y, Liu Y (2018). Time and job satisfaction: a longitudinal study of the differential roles of age and tenure. J Manag.

[18] Kim M, Park O, Oh S (2002). Nonlinear relationship between organizational tenure and job satisfaction: multi-level analysis. Kor Acad Organ Manag.

[19] Aleisa E, Al-Sobayel H, Buragadd S, Rao MG (2014). Rehabilitation services in Saudi Arabia: an overview of its current structure and future challenges. J Gen Practice.

[20] Albougami AS, Almazan JU, Cruz JP, Alquwez N, Alamri MS, Adolfo CA, Roque MY (2020). Factors affecting nurses’ intention to leave their current jobs in Saudi Arabia. Int J Health Sci (Qassim).

[21] Istichomah I, Andika IP, Pesirahu HV (2021). Social support affect nurses' job satisfaction: a literature review. Open Access Maced J Med Sci.

[22] Deshmukh N, Raj P, Chide P, Borkar A, Velhal G, Chopade R (2023). Job satisfaction among healthcare providers in a tertiary care government Medical College and Hospital in Chhattisgarh. Cureus.

[23] Sodani PR, Nair KS, Agarwal K (2023). Health system financing: a comparative analysis of India and Saudi Arabia. J Health Manag.

[24] Gosseries O, Demertzi A, Ledoux D (2012). Burnout in healthcare workers managing chronic patients with disorders of consciousness. Brain Inj.

